# Machine learning algorithm as a sustainable tool for dissolved oxygen prediction: a case study of Feitsui Reservoir, Taiwan

**DOI:** 10.1038/s41598-022-06969-z

**Published:** 2022-03-07

**Authors:** Balahaha Fadi Ziyad Sami, Sarmad Dashti Latif, Ali Najah Ahmed, Ming Fai Chow, Muhammad Ary Murti, Asep Suhendi, Balahaha Hadi Ziyad Sami, Jee Khai Wong, Ahmed H. Birima, Ahmed El-Shafie

**Affiliations:** 1 Department of Civil Engineering, College of Engineering, Universiti Tenaga Nasional (UNITEN), 43000 Kajang, Selangor Malaysia; 2 Civil Engineering Department, College of Engineering, Komar University of Science and Technology, Sulaimany, Kurdistan Region 46001 Iraq; 3 Discipline of Civil Engineering, School of Engineering, Monash University Malaysia, Jalan Lagoon Selatan, 47500 Bandar Sunway, Selangor Malaysia; 4 School of Electrical Engineering, Telkom University, Bandung, Indonesia; 5 Department of Civil Engineering, College of Engineering, Qassim University, Unaizah, Saudi Arabia; 6 Department of Civil Engineering, Faculty of Engineering, University of Malaya, 50603 Kuala Lumpur, Malaysia; 7 National Water and Energy Center, United Arab Emirates University, Al Ain, United Arab Emirates

**Keywords:** Hydrology, Natural hazards

## Abstract

Water quality status in terms of one crucial parameter such as dissolved oxygen (D.O.) has been an important concern in the Fei-Tsui reservoir for decades since it’s the primary water source for Taipei City. Therefore, this study aims to develop a reliable prediction model to predict D.O. in the Fei-Tsui reservoir for better water quality monitoring. The proposed model is an artificial neural network (ANN) with one hidden layer. Twenty-nine years of water quality data have been used to validate the accuracy of the proposed model. A different number of neurons have been investigated to optimize the model's accuracy. Statistical indices have been used to examine the reliability of the model. In addition to that, sensitivity analysis has been carried out to investigate the model's sensitivity to the input parameters. The results revealed the proposed model capable of capturing the dissolved oxygen's nonlinearity with an acceptable level of accuracy where the R-squared value was equal to 0.98. The optimum number of neurons was found to be equal to 15-neuron. Sensitivity analysis shows that the model can predict D.O. where four input parameters have been included as input where the d-factor value was equal to 0.010. This main achievement and finding will significantly impact the water quality status in reservoirs. Having such a simple and accurate model embedded in IoT devices to monitor and predict water quality parameters in real-time would ease the decision-makers and managers to control the pollution risk and support their decisions to improve water quality in reservoirs.

## Introduction

Reservoirs water considers one of the most crucial sources for household needs, irrigation, and other purposes such as industrial needs^1^. However, reservoir's water quality is susceptible to deterioration^2^. The reservoir's water quality status is measured based on three different properties such as physical, chemical, and biological^3,4^. Various water quality parameters are measured for each mentioned property to evaluate water quality. Therefore, there is a need to accurately model these parameters due to their importance for better management and mitigating any risk associated with sustaining the quality within the acceptable level^5^. Dissolved Oxygen (D.O.) is among the most critical parameters in measuring water quality status^6^. Among all the water quality parameters, the Dissolved Oxygen (D.O.) is considered the most representative parameter that showed the class's water quality status, especially in surface water. This is due to the fact that D.O. is vital for the aquatic organisms and fish in the water bodies. The level of dissolved oxygen is a reflection of wind and aerating action. The D.O. level must be within amount to assure the stability of organisms and fish life in the water bodies; the higher the D.O., the better the condition aquatic and fish survival. To indicate the state of any aquatic system, D.O. is used as an indicator, and it is essential for microorganisms when its present in water column^7^.

Deterministic and stochastic models are used to model the D.O. concentration changes and capture any pattern from the measured data; However, these models require massive data to model the D.O. pattern and consider very complex^8^. Other models, such as the statistical model introduced to overcome the conventional models. Since many factors impact the concentration of D.O. in the reservoir, which can cause to nonlinearity pattern, the statistical model fails to capture it since it assumes that the relationship between D.O. and other parameters is linear^9^. Alternatively, Machine Learning (ML) techniques have been proposed as an other technique to capture the nonlinearity in any complex system^10,11^.

Artificial Neural Network (ANN) methods were used in conjunction with numerical simulation models to boost the simulation results^12^. Recently, ML techniques have been used intensively in modeling complex parameters related to water resources, such as predicting sea-level rise^13–15^, rainfall prediction^16,17^, reservoir water level prediction^18,19^, and streamflow forecasting^11,20,21^. Inspired by the robust performance of ML in capturing the nonlinearity patterns in most of the engineering systems, different algorithms of ML have been adopted to predict the water quality parameters. Predicting the class Water Quality Index (W.Q.I.) has been carried out using different ML algorithms by many researchers^22–24^. Artificial Neural Network (ANN) has been used to predict total nitrogen and phosphorus in the United States (U.S.) lakes^25^. At the same time, a support vector machine (SVM) was developed to predict the concentration of biological oxygen demand (B.O.D.) at the Johor river, Malaysia^26^.

Regarding dissolved oxygen concentrations, an adaptive neuro-fuzzy inference system (A.N.F.I.S.) was proposed to predict D.O. at the Johor river, Malaysia^8^. However, the limitations of the A.N.F.I.S. model were reported by Ahmadlou et al.^27^. These drawbacks are that it is not very accurate and cannot find the best parameters; it is also prone to get stuck in a local minimum, contributing to its lack of prediction abilities.

A model was developed by Heddam^28^ to predict dissolved oxygen concentration using an optimally pruned extreme learning machine (O.P.E.L.M.). The study found that O.P.E.L.M. provided a reasonable estimate of D.O. However, Sánchez-Monedero et al.^29^ found that O.P.E.L.M. tends to degrade too many neurons, which results in noticeable performance degradation in some data sets.

The least-squares support vector (L.S.S.V.R.) has been proposed by Liu et al.^30^ to predict the amount of dissolved oxygen in intensive anaerobic ponds. However, It has been found that L.S.S.V.R. performance depends heavily on selecting the kernel coefficient and regularization coefficient, which are necessary for the optimization process and the final L.S.S.V.R. model. Regrettably, there is no unique, perfect method to specify the given parameters in the L.S.S.V.R. model. Extreme machine learning was developed by^31^ and used to predict dissolved oxygen in urban rivers. In addition to that, recently, the concentration of dissolved oxygen in fishery pond was predicted using a gated recurrent unit^32^.

To overcome the inherent limitations established by standalone models, hybrid models have been proposed to optimize these algorithms' hyper-parameters by augmenting them with different optimization algorithms. For instance, different hybrid models have been developed and used to predict dissolved oxygen concentration^33–35^. Teaching–learning-based optimization algorithm (T.L.B.O.) is used to predict dissolved oxygen^36^. Various regression equations were optimized, including quadratic, exponential, logarithmic, and linear using T.L.B.O. Then the findings from T.L.B.O. compared with an artificial bee colony (A.B.C.) optimizer. Better results were obtained by hybridizing the quadratic regression equation with T.L.B.O. Besides the hybridized model's complexity, the authors used many parameters (twenty parameters) as inputs to develop the model. One of the drawbacks of such a model is to have access to a significant amount of available and reliable water quality parameters data, which is challenging.

Despite the acceptable performances these models achieved, however, there are few limitations associated with the hybridization of ML. One of these limitations is the complexity and complicated architecture and the difficulties in initializing the input parameters for these hybrid models^37^. Kumar et al.^38^ found that the artificial neural network's prediction performance can be enhanced by improving the training approach without hybridizing it with optimization algorithms. In addition to that, a recent study highlighted the importance of the input combinations of ML algorithms' output accuracy, where the optimal input combinations can lead to a high level of accuracy without the need to augment ML with optimizers^39,40^.

Therefore, this study's chief aim is to propose an artificial intelligence model with simple architecture and a high-performance level to predict dissolved oxygen concentrations. This study will use historical data recorded for 29 years from the Fei-Tsui Reservoir to train the model to accomplish this goal. The number of neurons will be optimized in order to obtain the desired results. Different input combinations will be investigated and examined to enhance the model's performance. Recently many researchers have been developing AI models with a few inputs^41^. For example, Moghadam et al. used four input parameters and DO concentration to predict DO concentration in three different lead times^42^. Therefore, in this study few input parameters will be investigated.

Sensitivity analysis and uncertainty analysis will be carried out to validate the proposed model. Different statistical indices will be introduced to inspect the proposed model performance. For better visualization, Taylor's diagram, violin plot, and percentage of relative error between the projected data and the observed one have been implemented in this study.

## Methodology

### Study area and data description

**Figure 1:**
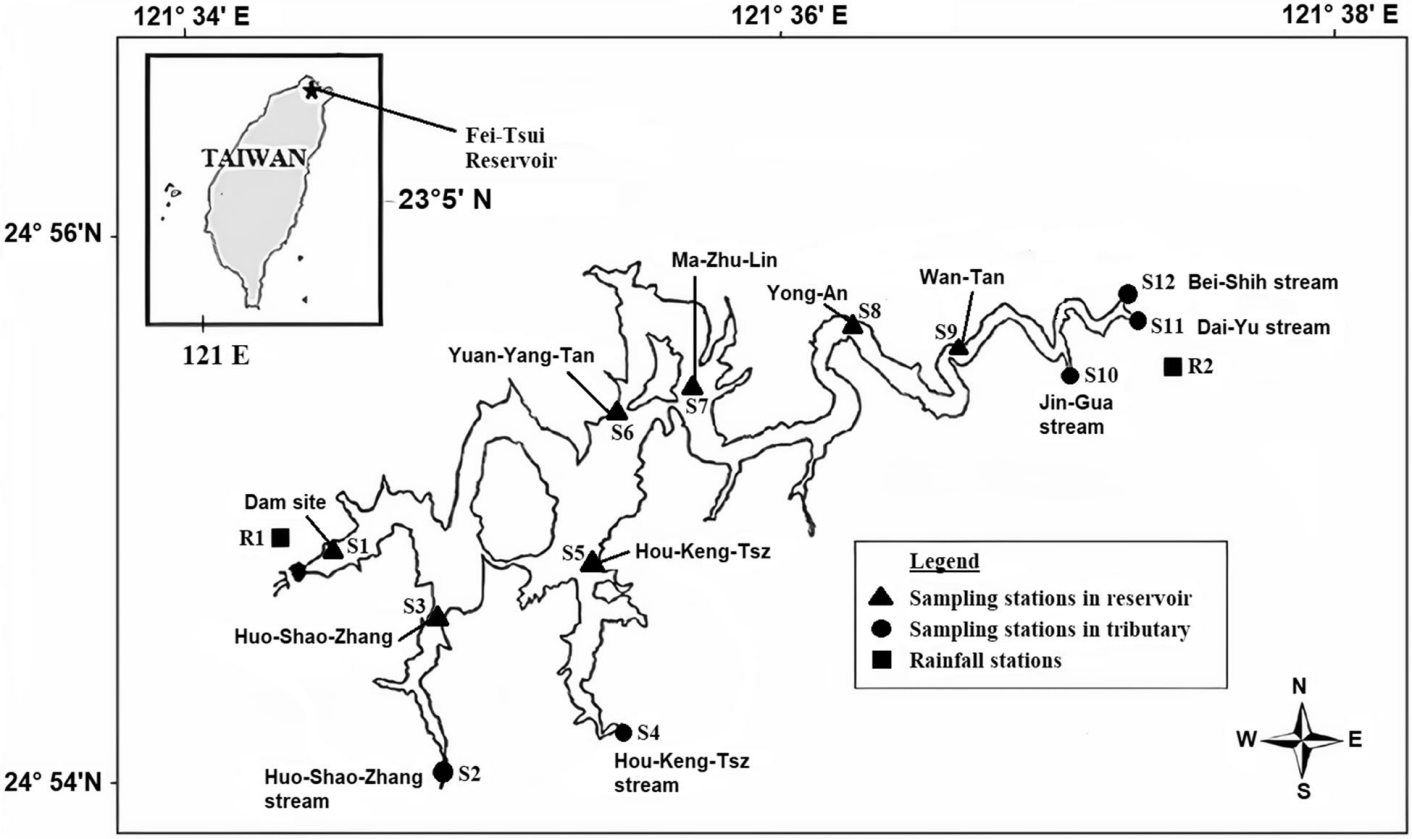
Location of Fei-Tsui Reservoir and sampling sites.

Located in Taiwan's north region, the Fei-Tsui reservoir serves a 300 km^2^ catchment area approximately, as shown in Fig. 1^43^. Since the 1980’s, for Taipei city, the Fei-Tsui reservoir is considered the primary source of drinking water. One hundred fifty days is the approximate duration when the water resides in the reservoir. Since 1987, monthly measurements have been conducted to examine the reservoir's water status based on different water quality parameters. The water quality samples have been collected at the outlet of the five inflow tributaries of the reservoir and another seven sampling locations at the reservoir's main lake^44^. The data was obtained from the administration office of the Taipei Fei-Tsui Reservoir. Table 1 shows the descriptive analysis of the measured Dissolved Oxygen (D.O.) concentrations.

**Table 1:** Descriptive analysis of the observed dissolved oxygen (D.O.)

Mean	7.91
Standard error	0.10
Median	8
Mode	8
Standard deviation	0.58
Sample variance	0.34
Kurtosis	15.27
Skewness	− 3.35
Range	3.41
Minimum	5.27
Maximum	8.68
Sum	229.66

### Model development

**Figure 2:**
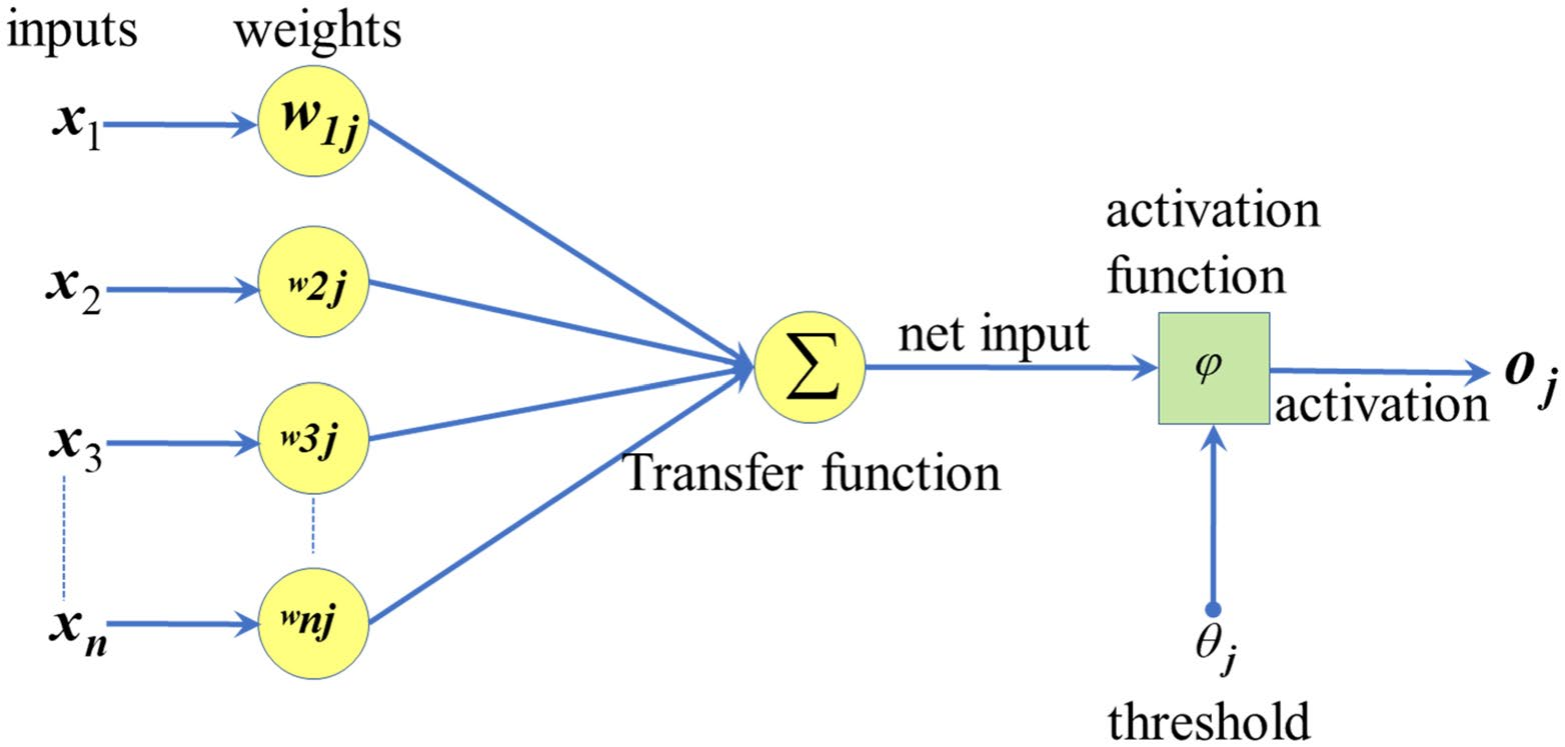
Structure of the proposed model*.*

An Artificial Neural Network with a single hidden layer was proposed to predict the dissolved oxygen concentration in the Fei-Tsui reservoir. The architecture of the proposed model can be seen in Fig. 2^45^. The proposed model consists of an input layer, which presents the input parameters that will be used to develop the model. In contrast, the output layer presents the model's output, which is the dissolved oxygen concentrations. Weights and biases connect the input and output layers to the hidden layer.

The hidden layer consists of several neurons. In this study, a different number of neurons will be investigated. In the beginning, the number of neurons will be set to equal five, then ten, fifteen, and finally, twenty. The predicted dissolved oxygen concentration will be compared with the observed concentration to choose the best model with the best-optimized number of neurons that give the lowest error. 29 years of monthly water quality data (348) will be used in developing the proposed model. 80% of the data will be used to train the model, while 20% will be used to test the model's accuracy. The pre-processing step was carried out by scaling the dataset between 0 to 1. Different types of activation functions and transfer functions will be explored and optimized.

Similarly, different training algorithms will be investigated to develop a model with a high level of precision. Finally, cross-validation with different k-fold (3, 5, 7 and 9) will be carried out to minimize the risk of overfitting. This validation procedure has been implemented using the trails-and-errors procedure, each trail with different value of the k-fold until achieving the one with minimal possibility of experienced overfitting. MATLAB Programming language was used to develop the proposed model.

One of the primary reasons for developing a model to predict the D.O. from other surface water parameters is that the D.O. is relatively costly and time-consuming to acquire and monitor. On the other hand, the main reason for selecting the Water Temperature, Biological Oxygen Demand, Iron, and Total Organic Carbon as a predictor for the D.O. is first because of the availability of these parameters. Secondly, there is a direct relationship between all these parameters and the D.O.; for example, the greater the amount of Biological Oxygen Demand in the water stream, the more rapidly is the depletion of the D.O. in water. Similarly, for the temperature (T), the more the T, the less the D.O. in the water stream will have occurred. Iron could critically consume the D.O. because of D.O. will be consumed as an oxidant for the Iron concentration. Hence, the D.O. concentration could dramatically reduce its amount in water stream. Finally, the Total Organic Carbon is the measuring indicator for how pure is the water stream considering the organisms’ life, which is indirectly affected by the level of D.O. in the water stream. Therefore, these parameters have been considered as predictors for D.O. in the current research. Table 2 shows the statistical analysis and the correlations between these parameters and D.O.

**Table 2:** Statistical analysis and coefficient of correlation between the input and the output parameters.

Parameters		Water temperature ℃	BOD mg/L	Iron mg/L	Total organic carbon mg/L
DO	Average	24.14	0.70	0.09	1.05
	Min	23.32	0.37	0.03	0.72
	Max	25.13	1.43	0.49	2.18
	Standard deviation (SD)	0.44	0.23	0.08	0.35
	Coefficient of variation (CV)	1.83	33.72	97.77	34.30
	Coefficient of correlation	− 0.49	0.46	0.17	0.27

**Figure 3:**
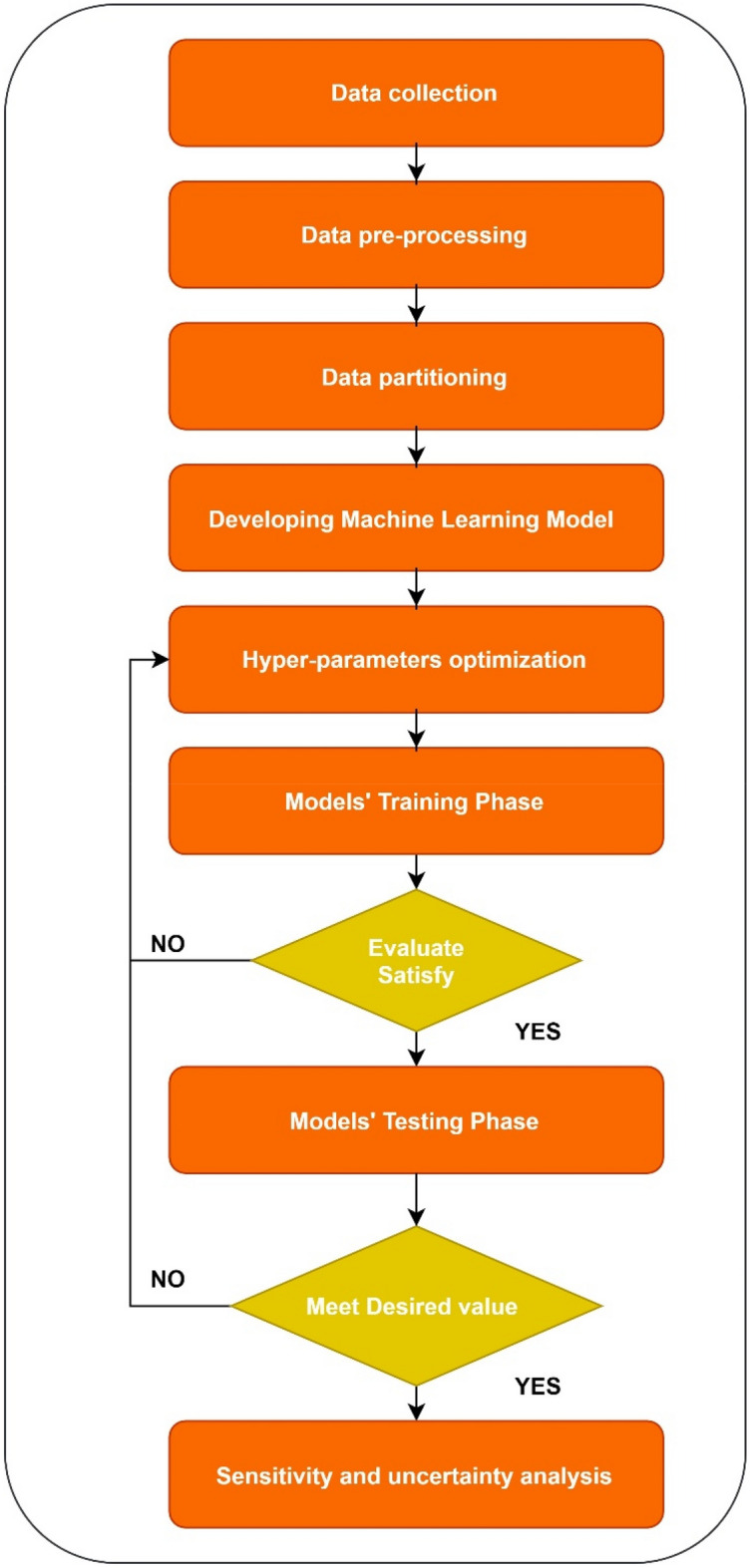
Flowchart of the study.

Three different statistical indices will be applied to measure how the proposed model predicts dissolved oxygen concentration. These indices are Root Mean Square Error (R.M.S.E.), Coefficient of Correlation (Correlation), and Coefficient of Determination (R-squared). The formulas for these indices with comprehensive explanation can be found in study carried out by Najah et al.^46^. In addition to that, Taylor's diagram and violin plots will be performed to assess the correlation between observed and predicted data. Sensitivity and uncertainty analysis will be carried to validate the proposed model's reliability. Figure 3 demonstrates the flow of the proposed method in this study. As can be seen from the flowchart, after the secondary data is collected, a pre-processing step was carried out to normalize the dataset before building the models. Then, different models will be built using different algorithms, and each model optimized by tuning the hyper-parameters of each model. In addition to that, a comparison between the proposed model and the developed models in literature will be carried out to highlight the contribution of this research.

## Results and discussion

One of the main characteristics of defining artificial neural network models is choosing the number of neurons for the hidden layer. An insufficient number of neurons can cause the model not to capture the data's nonlinearity^47^. However, if more neurons are introduced, that might increase the model's time and lead to overfitting. Therefore, to overcome such issues, in this study, different models were developed to find the optimum number of neurons where the number of neurons set to be 5, 10,15, and 20. In addition to that, identifying the right input combination is one of the vital factors that need to be considered in optimizing the performance of the artificial neural network model^5,26,48^. In this study, five different models with different input combinations have been introduced. The first model (M.1) was developed using one input parameter (water temperature), while the second model (M.2) was developed by introducing another input parameter (water temperature and biological oxygen demand). A third model (M.3) used three input parameters (biological oxygen demand, iron, and total organic carbon). The fourth model (M.4) was developed using a different combination of three parameters (water temperature, biological oxygen demand, and iron). And finally, model five (M.5) was developed using all four parameters as input (water temperature, biological oxygen demand, iron, and total organic carbon). Twenty models have been developed with different inputs and numbers on neurons to find the best model for predicting dissolved oxygen concentrations changes. Table 3 presents the performance of each developed model for the optimum number of neurons. It can be seen that the best number of neurons falls in the range of 5 to 15 for each model. It can be seen, in all the proposed models, the number of neurons contributes significantly to the improvement of the models’ accuracy, except in model five (M.5). It has been noticed that the poor performance in M.5 is associated with the input combinations, not with the number of neurons. Such findings reveal the input combinations' importance in developing a robust model. In addition to that, it can be observed from Table 3 that the best performance model (M.4) can be achieved when the number of neurons is equal to 15 and the combination of the input is water temperature, biological oxygen demand, and iron. This is followed by M.2 with the same number of neurons but with two input combinations (water temperature and biological oxygen demand). It can also be observed that when the total organic carbon was introduced as input in M.3 and M.5, the artificial neural network models' accuracy dropped. This indicates that the total organic carbon should not be considered input in developing reliable models to predict dissolved oxygen concentration changes. Feed-forward MLP model is used in this study. Regarding the training algorithm, three different algorithms were investigated, namely Levenberg–Marquardt, Bayesian regularization and Scaled conjugate gradient. The best results obtained by using the latter training algorithm. Scaled conjugate gradient is powerful training algorithm where there is no need for much memory. It is also proved to be faster in convergence compared to the other two used training algorithms. With regards to activation function, it was found that tanh (hyperbolic) function is best among the different inspected functions.

**Table 3:** Performance of each developed model based on the optimum number of neurons.

Number of neurons	10	15	5	15	10
Models	**M.1**	**M.2**	**M.3**	**M.4**	**M.5**
Correlation	0.929	0.971	0.865	**0.988**	0.598
R-squared	0.857	0.940	0.731	**0.980**	0.336
RMSE	0.240	0.143	0.293	**0.136**	0.487

Significant values are in bold.

It can be seen from Table 4 the performance of each developed model with a different input combination. It can be observed that the fourth model (M.4) outperforms all other models in predicting the dissolved oxygen and manages to capture the peak and low concentration of the dissolved oxygen. Moreover, it can be seen that the mean of the predicted data is close to the actual observed data.

**Table 4:** Comparison between the proposed model and the actual DO for the testing dataset.

	Actual	M.1	M.2	M.3	M.4	M.5
Max	**8.680**	8.560	8.747	8.680	**8.386**	8.772
Mean	**7.920**	8.008	7.939	7.890	**7.816**	8.024
Min	**5.270**	5.270	5.302	5.972	**5.149**	7.509
SD	**0.577**	0.601	0.596	0.535	**0.579**	0.235

Significant values are in bold.

**Figure 4:**
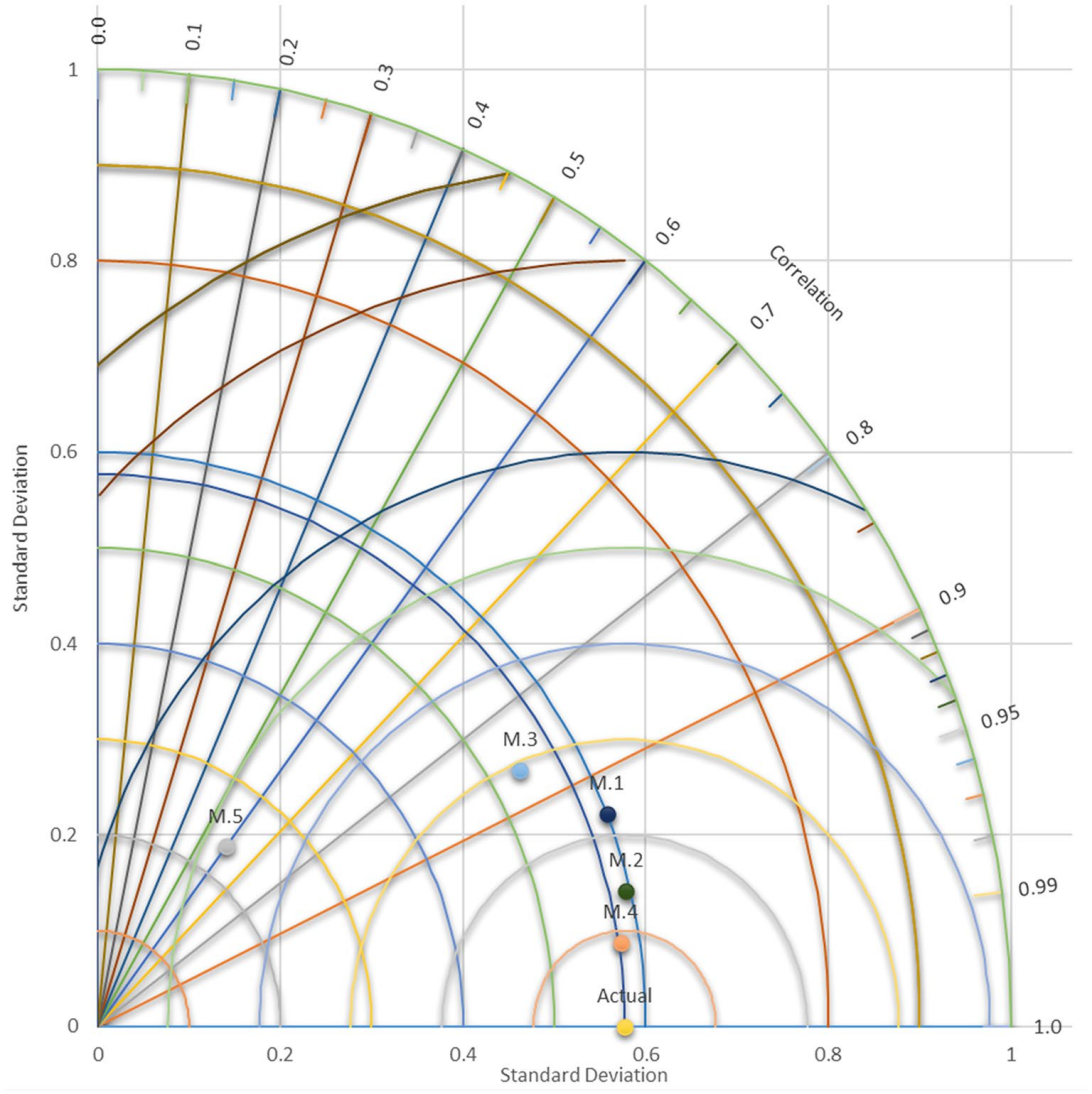
Taylor diagram for the proposed five models.

To test the proposed model's reliability and to determine the model's validity, Taylor’s diagram is recommended by many researchers and is commonly used^19,49^. It can be seen from Fig. 4 the relation between the correlation and the standard deviation for the actual and the predicted concentration of dissolved oxygen for the five models. It can be seen that M.4 is outperforming all other models where the distribution of standard deviation for the predicted data is close to the actual one, which suggests that the proposed model is consistent in capturing the observed data pattern.

**Figure 5:**
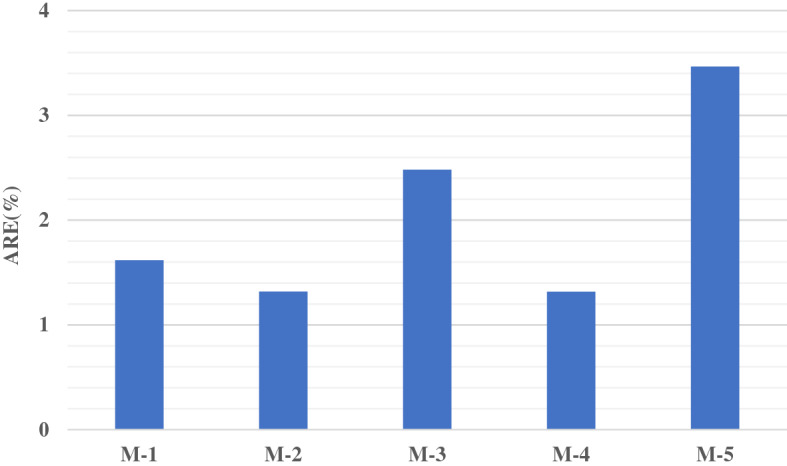
Average Relative Error of each proposed model.

The average percentage of relative error for each model has been computed to examine the error percentage between the predicted and the actual observed data, as shown in Fig. 5. The value confirmed this study, where M.4 indicates the lowest error compared to other developed models. While M.2 is ranked second, and the highest error observed with M.5.

**Figure 6:**
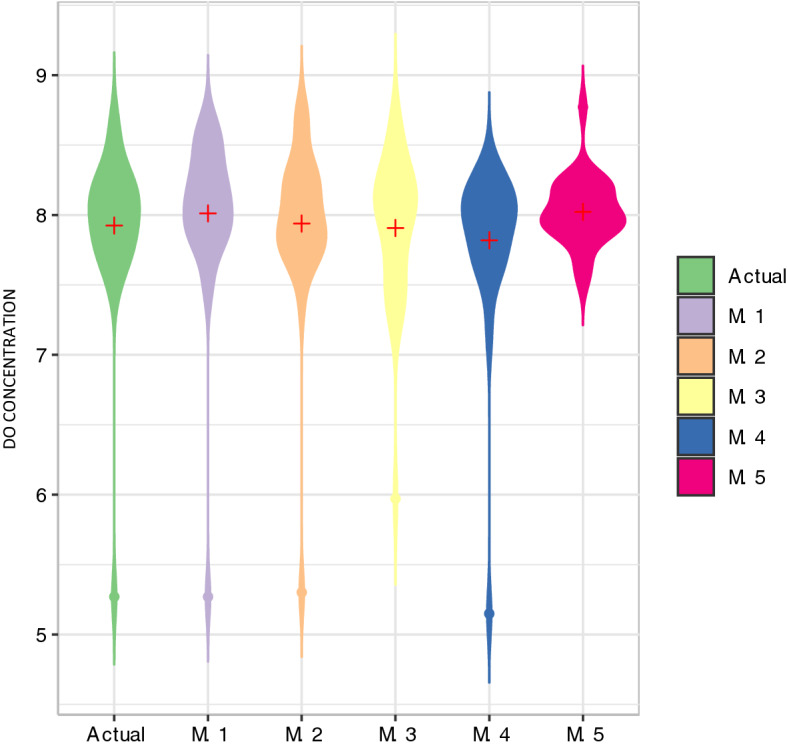
Violin plot between actual and proposed models.

A Violin plot is used to demonstrate the difference between the actual and predicted data from each model, as shown in Fig. 6. This plot helps to understand the probability distribution of the data. It can be seen that the best model is M.4, which its predicted data have similar distribution compared with the actual data.

To measure the proposed models' performance when new data are introduced, the d-factor value is used for this purpose. When the d-factor values close to zero mean that the model can still perform well if a new data set is introduced^50^. This study uses the following equations to calculate d-factor values:

1 $$\text{d{-}factor}=\frac{{\overline{\mathrm{d}} }_{\mathrm{x}}}{{\upsigma }_{\mathrm{x}}}$$

2 $${\overline{\mathrm{d}} }_{\mathrm{x}}= \frac{1}{\mathrm{N}} \sum_{\mathrm{i}=1}^{\mathrm{N}}\left({\mathrm{X}}_{\mathrm{U}}-{\mathrm{X}}_{\mathrm{L}}\right) \quad \mathrm{i }= 1, \ldots ,\mathrm{N}$$

**Figure 7:**
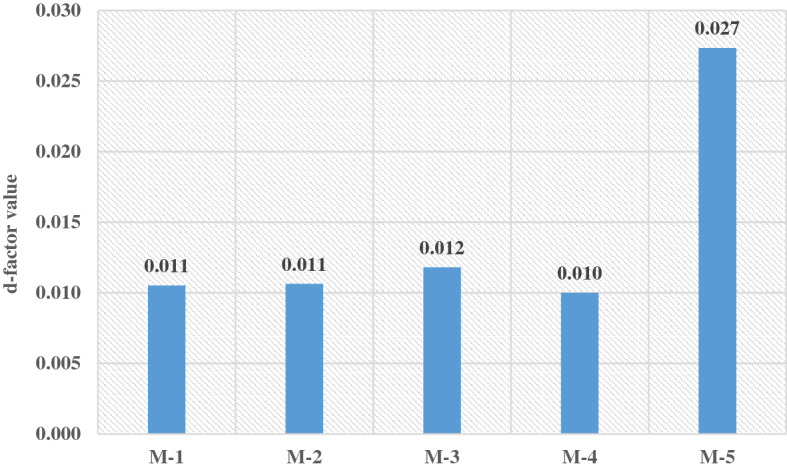
d-factor values for each proposed model.

${\upsigma }_{\mathrm{x}}$ represents the standard deviation of actual data x and ${\overline{\mathrm{d}} }_{\mathrm{x}}$ represents the average distance between the upper ${\mathrm{X}}_{\mathrm{U}}$ (the value that is greater than or equal to every element in the dataset) and lower ${\mathrm{X}}_{\mathrm{L}}$(the value that is less than or equal to every element in the dataset), i denotes the order of the record in the time series data (i = 1,…,N), while N represents the number of the observed dataset. It can be seen from Fig. 7 that M.4 shows the lowest d-factor value, which indicates this model architecture is reliable to be adopted when a new set of data used and can perform with a high level of accuracy.

**Figure 8:**
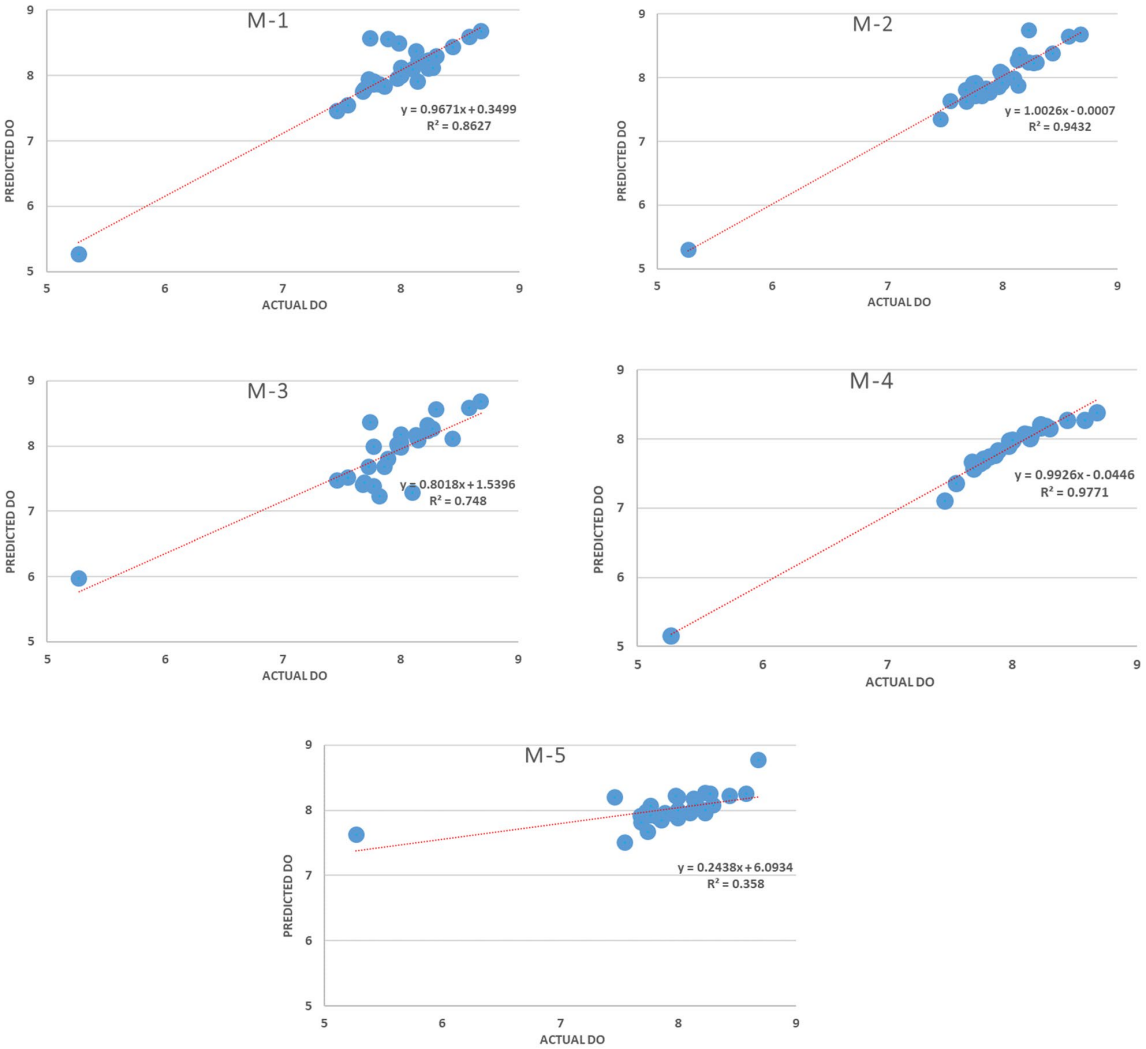
Predicted vs. actual scatter chart.

Figure 8 shows the scatter plots between the predicted and actual D.O. for the five developed models where it can be seen M.4 outperformed all other models with different input combinations.

To sum up, the lowest accuracy reported when M.5 used to predict D.O. It should be noted that M5. model exhibits acceptable accuracy in capturing the maximum and average values of D.O. concentrations. However, for the minimum values of D.O. concentrations, the M.5 model is unable to capture it where the absolutes relative error percentage ranged between 42 to 59%. Model M.3 ranked four among the five developed models with an average relative error percentage of 5%. M.1 ranked three among the five other models. It performs better than M.3 and M5 where the average absolute relative error percentage equals 1.7%. And finally, M.2 ranked second, outperforming M.1, M.3, and M.5. However, M.2 was unable to capture the extreme concentration values of D.O. as M.4.

A comparison was conducted between the proposed model and other literature models to compare the current study findings to other studies. Kisi et al.^51^ proposed a Bayesian model averaging (B.M.A.) model to predict the concentration of dissolved oxygen. And the findings were compared with different data-driven methods, including an extreme learning machine (E.L.M.), classification and regression tree (CART), and adaptive neuro-fuzzy inference system (A.N.F.I.S.). The r-squared for testing ranged from 0.718 to 0.836 (B.M.A. (0.836), E.L.M. (0.822), A.N.F.I.S. (0.831) and CART(0.718)) for one of the stations used in the study. Four input parameters were used as inputs to develop the four models. In the current study, only three parameters were used as input to the model. As mentioned earlier, choosing the right combinations has a crucial impact on the performance of the model. In the current study, when water temperature, biological oxygen demand, and iron were used as input, the artificial neural network achieved a high accuracy level where r-squared equals 0.98. Multi-layer perceptron neural network developed to predict dissolved oxygen concentration in Malaysia's Johor River^52^. In this study, five different input combinations were used to develop the model. The performance of the model was acceptable were r-squared was equal to 0.95. Compared with the proposed model in the current study, a high accuracy level has been achieved where r-squared equals 0.98 with a smaller number of input combinations. It can be concluded that the current proposed model is more accurate and can be adopted as a tool to predict the changes in the concentration of dissolved oxygen. A point that can be raised from the earlier comparison is that the studies were conducted using different datasets in different countries. Therefore, for future works, a more valid comparison should be performed to consider these algorithms in predicting dissolved oxygen concentrations at the Fei-Tsui reservoir.

For comparison purposes, the performance of the developed model (M.4) was compared with two other models, namely Random Forest (R.F) and Boosted Tree (B.T) regressions. The comparison was carried out using maximum and average relative percentage error. It has been observed that the maximum relative percentage error for M.4 is equal to 4.7%, while the maximum relative percentage error for B.T and R.F is 46% and 49%, respectively. At the same time, the average relative percentage error for M.4 is 1.3% which is the lowest than both B.T (4.1) and R.F (4.6).

## Conclusion

The study focuses on predicting dissolved oxygen concentration as crucial water quality parameters in the Fei-Tsui reservoir in Taiwan using an artificial neural network model with simple architecture. Twenty-nine years of historical data provided the basis for development of the model. To test the model's reliability and optimize the algorithm, different numbers of neurons were used. Various numbers of input combinations were used to enhance the model's accuracy. Statistical indices were used to validate the accuracy of the model. The results reveal that the best number of neurons equals fifteen, while the best input combinations are three input parameters. These parameters are water temperature, biological oxygen demand and iron. The proposed model exhibits a high level of accuracy in predicting dissolved oxygen concentration changes where the r-squared is equal to 0.98. Taylor's diagram shows that the proposed model (M-4) displays a high consistency and accuracy level. Further investigation in implementing the proposed model in this research can predict other water quality parameters and be applied at locations with different climatic conditions for generalization purposes. There is a need to investigate machine learning models' integration with sensing technologies to efficiently monitor and predict water quality parameters for a smart early warning system. In addition, although the proposed optimization of the hyper parameters of the ANN modeling approach could provide proper prediction accuracy for DO, the accuracy could be improved by implementing the optimization of the hyper parameters of other AI model such as Random Forest and Boosted Tree algorithm.
